# One-stage hybrid operation for hypervascular central nervous system tumors: a single-center experience of 31 cases

**DOI:** 10.1186/s41016-025-00400-y

**Published:** 2025-07-31

**Authors:** Mingze Wang, Zhikang Zhao, Shuo Wang, Yong Cao, Jizong Zhao

**Affiliations:** 1 Department of Neurosurgery, Beijing Tiantan Hospital, Capital Medical University, Beijing, 100070 China; 2 China National Clinical Research Center for Neurological Diseases, Beijing, 100070 China; 3 Center of Stroke, Beijing Institute for Brain Disorders, Beijing, 100069 China

**Keywords:** Hypervascular tumors, Central nervous system, One-staged hybrid operation, Endovascular embolization, Microsurgery

## Abstract

**Background:**

Surgical resection for hypervascular central nervous system tumors poses a significant challenge for neurosurgeons. Controversy remains about the effect and safety of the traditional therapeutic mode, which combines preoperative embolization and delayed tumor resection, remain controversial. Whether a one-stage hybrid operation modality offers a novel approach to address treatment challenges in a safer and more effective way remains unknown.

**Methods:**

From the neurosurgical operation database, we retrospectively reviewed patients with hypervascular central nervous system tumor patients who underwent one-stage hybrid operation between January 1, 2014, and September 30, 2024. Intraoperative blood loss, the percentage of tumor devascularization, and complications associated with embolization were recorded. Novel embolization strategies used to facilitate the resection of tumors in one-stage hybrid operations were analyzed.

**Results:**

In total, 31 hypervascular central nervous system tumor patients were recruited. The main pathological types included various types of meningiomas (45.2%), hemangioblastomas (16.1%), paragangliomas (9.7%), and solitary fibrous tumors (9.7%). Embolization of tumor-feeding arterial pedicles alone was achieved in 25 patients, and various materials, such as ethylene–vinyl alcohol copolymer, Guglielmi detachable coil, and silk suture segments, were used, in which the tumor blood supply was blocked satisfactorily and the texture became softer postembolization. Intratumoral vascular beds were embolized in six patients. The mean occlusion rate of the target pedicle was 83.3%. Gross-total resection was achieved in 22 patients (71.0%), with a mean blood loss volume of 1127 ± 1114.4 mL (ranging from 150 - 4500 ml). No embolization-related complications occurred. Deterioration of neurological deficits was observed in three patients (9.7%) at discharge.

**Conclusion:**

A one-stage hybrid operation is safe for the treatment of hypervascular central nervous system tumors. A prospective study to evaluating its safety and efficacy compared with separate-stage treatment is needed.

## Background

Hypervascular central nervous system (CNS) tumors represent a significant clinical challenge in neurosurgical practice, primarily because of their dense vascular networks, which frequently precipitate catastrophic intraoperative hemorrhage. The proliferative angiogenesis inherent to these lesions not only complicates surgical visualization by obscuring tumor margins but also heightens the risk of iatrogenic neurological injury during resection. Preoperative embolization has emerged as a potential adjunct to mitigate perioperative morbidity, with reports suggesting its efficacy in reducing intraoperative blood loss [1–3], even in lesions mimicking arteriovenous malformations (AVMs) in their vascular complexity [4]. However, the definitive efficacy of this intervention remains contentious. While some studies advocate its role in minimizing hemorrhagic risks and facilitating gross-total resection, others underscore the lack of robust evidence corroborating these outcomes [5, 6]. Furthermore, postembolization complications are recognized as pressing issues that require attention [7]. There is an urgent need for an innovative solution that can achieve both vascular obliteration and elimination of complications.

In recent decades, the advent of one-stage hybrid neurosurgical procedures has catalyzed a paradigm shift in cerebrovascular and oncological interventions, redefining therapeutic strategies through the integration of endovascular and open surgical techniques [8]. Encouraged by their established efficacy in treating complex cerebrovascular pathologies, such as arteriovenous malformations and aneurysms, hybrid approaches have increasingly garnered interest for managing hypervascular CNS tumors, where hemostatic control and tumoral vascular obliteration are paramount. While preliminary investigations involving small cohorts have demonstrated feasibility and safety [9, 10]. In this report, we present our experience with the use of a one-stage hybrid operation for the treatment of hypervascular tumors, and discuss several novel embolization strategies that we used to facilitate the resection of tumors in one-stage hybrid operations.

## Methods

### Aim

To evaluate the safety and efficiency of one-stage hybrid operations in the treatment of hypervascular tumors of the CNS.

### Study design and participants

A retrospective analysis was conducted on a neurosurgical operations database spanning January 1, 2014, to September 30, 2024. Among 92,255 patients who underwent tumor resection procedures over this 10-year period, 86 were identified as having received preoperative endovascular intervention followed by tumor resection for hypervascular CNS tumors. Notably, 31 of these patients underwent combined endovascular and microsurgical procedures as a single-stage hybrid operation in a dedicated hybrid operating room (OR), integrating real-time imaging and surgical intervention.The Ethics Committee of Beijing Tiantan Hospital has reviewed and approved this study (KY2016-034-02).

Patients were reviewed and selected with the following inclusion criteria: (1) Definite pathological diagnosis of an occupying tumor of the CNS; (2) remarkable abnormal abundance of blood vessels within the tumor tissue, including contrast enhancement on computed tomography (CT) or magnetic resonance imaging (MRI) scans, a vascular pattern within the tumor, and increased blood flow within the tumor; and (3) receiving both endovascular and microsurgical/endoscopic procedures in the hybrid OR. Patients who met the following criteria were excluded: (1) Receiving endovascular and microsurgical/endoscopic procedures in separate operations or in the same operation but with the transportation of the patient, and (2) endovascular and microsurgical/endoscopic procedures were performed on different lesions, or unrelated treatment was performed.

### Workflow of one-stage hybrid operation

The workflow for one-stage hybrid operations targeting hypervascular CNS tumors is tailored to individual patient needs, emphasizing seamless integration of endovascular and surgical techniques. The defining feature of this approach is the synchronous execution of endovascular interventions—such as feeding arterial embolization or balloon-assisted temporary occlusion—with microscopic or endoscopic tumor resection, enabling real-time hemostatic control and tumor debulking. Key components of the workflow include meticulous preoperative multidisciplinary planning and precise intraoperative implementation, ensuring alignment between procedural goals and patient-specific anatomical and vascular considerations.

In the preoperative discussion, every candidate was discussed by multidisciplinary experts for his/her indication and technical details of the operation. Patients who met the following criteria were considered candidates: (1) With neuroimaging evidence of a rich blood supply, such as the appearance of flow-void or pepper-salt signs on MRI and pronounced staining on digital subtraction angiography (DSA); (2) with a tumor having a large volume and located in surgically challenging areas such as the skull base, foramen magnum, and jugular foramen; (3) with a tumor supplied by blood vessels originating from deep within the surgical field, making it difficult to control the bleeding and safely perform piecemeal resection of the tumor; and (4) with a need for preoperative embolization but had the second pathobiological process, such as ischemic necrosis, edema, and subsequent cerebral hernia. Conclusions can be drawn about (1) the necessary preoperative preparation, (2) the surgical methods and approach for tumor resection, and (3) the shortcomings of surgical treatment that can be compensated for by interventional methods. The discussion determines the role division, treatment goals, and technical details of the interventional surgery and surgical procedures. Notably, the objective of endovascular embolization is not to achieve maximal devascularization but rather to occlude arterial pedicles that are inaccessible for microsurgical or endoscopic manipulation.

### Strategy of one-stage hybrid operation

The operation was performed in a one-stage hybrid OR, which essentially consisted of a surgical microscope (Pentero^®^900, Carl Zeiss Surgical AG, Oberkochern, Germany), a radiolucent operating table (MAQUET Holding B.V. & Co. KG, Rastatt, Germany), and a monoplane angiography unit. All three monoplane angiography units mounted in our three hybrid ORs are competent for this work, including the ARTIS Pheno and ARTIS Zeego systems (Siemens Healthineers, Erlangen, Germany) and the Allura Xper FD20 system (Philips, Netherlands).

## Endovascular session

The implementation of the endovascular procedure should adhere to the following considerations:

(1) Embolization should prioritize the arterial feeder that is difficult to access surgically (the target feeding arteries). There is no need to embolize superficial arterial feeders, as they can be managed through microsurgical or endoscopic techniques.

(2) The focus of embolization should be the main trunk of the peritumoral arterial feeders (alone, embolization of tumor-feeding arterial pedicles). It is not recommended to inject embolic agents directly into the tumor, as this could make the tumor harder and more difficult to resect.

(3) In cases where the target feeding arteries are difficult to access endovascularly, such as branches of the ophthalmic artery, the middle cerebral arterial trunk, or the vertebral arterial trunk, embolization can be considered from an accessible pedicle that allows the embolic agent to flow from the intra/peritumoral vascular network to inaccessible arterial feeders. Moreover, embolization of the intratumor vascular network, which makes tumors harder and increases the difficulty of resection, may be avoided. A compliant balloon system can be utilized for temporary occlusion of the main trunk to control uncontrollable massive bleeding during tumor resection.

(4) A compliant balloon system could also be utilized for temporary control of bleeding during craniotomy and tumor exposure.

The patient was placed under general endotracheal anesthesia in the supine position for the endovascular procedure. Femoral access was established via a 6-F sheath (AVANTI^®^ Introducer, Cordis Corporation, Miami Lakes, FL, USA) with a modified Seldinger technique. A 6-F guiding catheter (Envoy^®^, Codman division of Johnson & Johnson Medical Ltd., Wokingham, Berkshire, UK) was placed at the petrous segment of the internal carotid artery or the foraminal segment of the vertebral artery. A microcatheter (Marathon 1.3-F, eV3/Covidien, MN, USA; Excelsior SL-10, Stryker Neurovascular, CA, USA; or Headway-17, MicroVention, CA, USA; or Prowler Select Plus, Codman, Raynham, MA, USA) is advanced over a microwire (Synchro-10, Stryker Neurovascular, CA, USA; Synchro-14, Stryker Neurovascular, CA, USA; Synchro-2, Stryker Neurovascular, CA, USA; or Traxcess-14, MicroVention, CA, USA) into the target arterial pedicles under roadmapping. Microcatheter angiography was performed to confirm the feeding area before embolization. Ethylene–vinyl alcohol copolymer (EVOH, Onyx 18/34, eV3/Covidien, MN, USA) or 5–0 silk suture segment (W500, Ethicon, Inc., Somerville, NJ, USA) injected through the microcatheter under roadmapping until the feeding area of the targeted arterial pedicle is excluded from circulation. Guglielmi detachable coil (GDC) could be used to assist in stabilizing liquid embolic agents. The arterial pedicle could be as the target of embolization rather than the vascular network of the tumor, with the goal of controlling bleeding during subsequent surgical resection. The advantage of the use of 5–0 silk suture segments for embolization is that only the feeding arteries are embolized, and microcatheters can be reused. The catheter system and sheath can be withdrawn in most situations, as there is no need for re-examination with DSA. Embolization-related complications were screened for and monitored during the endovascular procedure. Hemorrhagic complications were identified through intraoperative cone-beam CT via C-arm fluoroscopy, whereas vascular occlusion was assessed using postprocedural angiographic imaging. Involvement of functional neurologic structures or cranial nerves was monitored via intraoperative electrophysiological monitoring.

## Microsurgical session

Patients were located in a surgical position as needed with their head fixed by a head frame. Surgical resections were conducted as planned via a microscope or endoscope. The craniotomies were performed via an appropriate approach. The feeding arteries, which were identified in the skin and dura but not embolized before the endovascular procedure, should be controlled as much as possible to reduce blood loss. Sometimes, a balloon catheter could be placed in the proximal trunk of the feeding artery to help control bleeding during skin incisions. When the tumor was exposed, the arterial feeders that were easy to access surgically were electrocoagulated following the tumor resection performed according to the results of prior embolization. If the feeding arteries of the tumor were largely blocked, piecemeal removal was performed.

### Outcome evaluation

Deterioration of neurological deficits (DNDs), intraoperative blood loss, occlusion rate of target arterial pedicles (occluding target arteries/all target arterial pedicles according to the preoperative embolization plan), and embolization-related complications were recorded as safety and efficacy outcomes. Embolization-related complication encompassed a spectrum of adverse events, including hemorrhagic complications (e.g., endovascular procedure-related hematoma) and ischemic complications (e.g., tumor infarction, edema, and inadvertent occlusion of critical vascular structures, functional areas, or cranial nerves). DND was defined as a modified Rankin Scale (mRS) score at discharge ≥ 3 and greater than that at admission.

### Data collection

The observational indicators collected included demographic information and medical history. Disease-related information included symptoms and signs, location and invasiveness of occupation on MRI, and vascular architectural findings on DSA. Treatment and outcome information included the type of hybrid operation, degree of tumor resection, pathological results, embolization-related complications, and neurological evaluation (using the mRS) at admission and discharge.

### Statistical analyses

All the statistical analyses were performed via IBM^®^ SPSS^®^ Statistics (Version 26, IBM, Armonk, NY, USA). The baseline data, disease-related characteristics, and treatment and outcome information were qualitatively and quantitatively described.

## Results

From the database of neurosurgical operations of Beijing Tiantan Hospital, 92,255 patients diagnosed with occupation of the CNS between January 1, 2014, and September 30, 2024, were retrieved. Eighty-six of the 92,255 patients received both endovascular intervention and tumor resection surgery for hypervascular tumors. Among these, 31 patients underwent endovascular and microsurgical procedures in a single session, that is, one-stage hybrid operation. The baseline information and imaging findings of the 31 enrolled patients are summarized in Table 1. For endovascular procedures, EVOH was used in 27 patients, EVOH + GDCs were used in three patients, and 5–0 silk suture segments were used in one patient. Different embolization modes were used: the embolization of major arterial pedicles was achieved in 25 patients (80.6%) and EVOH into the intratumoral vascular bed was achieved in six patients. The mean occlusion rate of the target pedicle was 83.3%. With the assistance of simultaneous endovascular embolization, gross-total resection was achieved in 22 patients (71.0%), near-total resection in six patients (19.4%), and partial resection in three patients (9.7%), with a mean blood loss volume of 1,127 ± 1,114.4 mL (ranging from 150 - 4500 ml). The details concerning the embolization procedure are summarized in Table 2. The mRS score remained unchanged in 27 patients (87.1%). DNDs were observed in three patients (9.7%). Other safety and efficacy outcomes, including intraoperative blood loss, complications associated with embolization in one-stage hybrid operations, neurological outcomes, and follow-ups, had been summarized in Table 3. Recurrence was observed in five cases, including two cases in 3 months with the pathological diagnoses of malignant peripheral nerve sheath tumor and paragangliomas, respectively, one case in 6 months with medulloblastoma and death in 12 months; and two cases in 12 months with the diagnoses of meningioma of endothelial type and anaplastic type respectively.

**Table 1:** Characteristics of baseline and neurovascular images

	One-staged group (*n* = 31)	
	*n*	Percentage
Age (years old, mean ± SD)	41.3 ± 15.97	
Male	17	54.8
Magnetic resonance imaging		
Largest diameter of tumor (cm, mean ± SD)	6.1 ± 2.22	
Tumor localization*		
Deep site of cerebrum	2	6.5
Superficial site of cerebrum	7	22.6
Cerebellum and stem	8	25.8
Anterior fossa	2	6.5
Middle fossa	5	16.1
Foramen magnum region	1	3.2
Jugular foramen	2	6.5
Cerebellopontine angle	3	9.7
Head and neck	4	12.9
Venous sinus invasiveness*		
Anterior 1/3 of superior sagittal sinus	2	6.5
Posterior 2/3 of superior sagittal sinus	4	12.9
Transversal sinus	1	3.2
Venous plexus in cervical and vertebral area	2	6.5
Flow-void sign	18	58.1
Digital subtraction angiography*		
Origin of arterial pedicles		
Internal carotid artery	8	25.8
External carotid artery	18	58.1
Vertebral artery	16	51.6
Branches of subclavian artery	2	6.5
Distinct venous drainage	7	8
Pathology		
Meningioma	14	45.2
Transitional type	3	9.7
Fibrous type	3	9.7
Endothelial type	2	6.5
Angiomatous type	1	3.2
Atypical type	1	3.2
Anaplastic type	1	3.2
Microcystic type	1	3.2
Unclassified type	2	6.5
Hemangioblastomas	5	16.1
Paragangliomas	3	9.7
Solitary fibrous tumor	3	9.7
Malignant peripheral nerve sheath tumor	2	6.5
Small cell malignancies of mesenchymal origin	1	3.2
Desmoid fibromatosis	1	3.2
Neurofibroma	1	3.2
Medulloblastoma	1	3.2
Acoustic neurilemoma	14	45.2

^*^A single lesion may involve multiple anatomic or vascular structures, resulting in a cumulative involvement rate that may exceed 100%

**Table 2:** Information of surgical resecting procedures

	One-staged group (*n* = 31)	
	*n*	Percentage
Embolizing material		
EVOH only	27	87.1
EVOH + GDC	3	9.7
5–0 silk suture segments	1	3.2
Embolization mode		
Pedicle only	20	64.5
Pedicle (dominant) + iTVB#	5	16.1
iTVB (dominant) + pedicle	2	6.5
iTVB only	4	12.9
Degree of resection		
Gross-total resection	22	71.0
Near-total resection	6	19.4
Partial resection	3	9.7

^#^*iTVB* intratumoral vascular bed

**Table 3:** Information of neurological outcomes

	One-staged group (*n* = 31)	
	*n*	Percentage
Volume of blood loss (ml)		
Mean ± SD	1127 ± 1114.4	
Median (IQR)	600 (400, 1500)	
Embolization-related complication	0	0
Modified Rankin Scale		
Admission		
0	8	25.8
1	12	38.7
2	4	12.9
3	4	12.9
4	2	6.5
5	1	3.2
Discharge		
0	7	22.6
1	10	32.3
2	4	12.9
3	6	19.4
4	3	9.7
5	1	3.2
Follow-up of 6 months		
0	21	67.7
1	2	6.5
2	3	9.7
3	3	9.7
4	1	3.2
5	1	3.2
Follow-up of 12 months		
0	20	64.5
1	2	6.5
2	5	16.1
3	2	6.5
4	1	3.2
5	0	0
6	1	3.2

### Case illustration

**Fig. 1:**
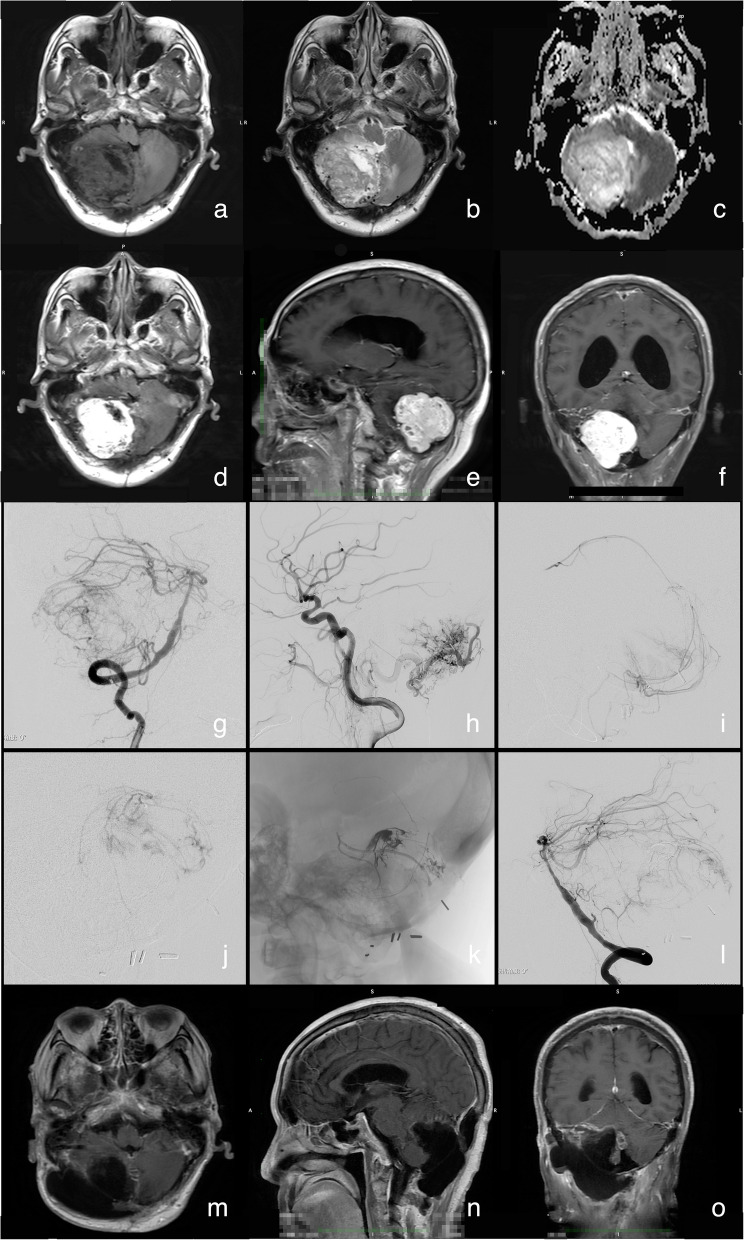
**a** and **b** MRI revealed postoperative changes in the posterior cranial fossa, with a mass-like ring-shaped signal-occupying lesion visible in the right cerebellar hemisphere. The borders of the lesion are ill-defined, and multiple vascular flow voids are observed around the white matter. The fourth ventricle is compressed and deformed, and the supratentorial ventricles are dilated. **c** Diffusion-weighted imaging revealed no significant diffusion restriction in the lesion. **d**, **e** In contrast imaging, the lesion demonstrated marked heterogeneous enhancement. **g** and **h** DSA revealed a hypervascular tumor in the right cerebellar hemisphere supplied by the right posterior inferior cerebellar artery, right superior cerebellar artery, right anterior inferior cerebellar artery, and dural branch of the right occipital artery. The tumor is drained by the superficial veins of the cerebellar hemisphere. **i** An angiogram via the microcatheter, which had superselected into the distal end of the posteroinferior cerebellar artery, verified the feeding of the tumor before embolization. **j** Microcatheter angiogram of the distal end of the anterior inferior cerebellar artery, which verified the supply of the tumor before embolization. **k** Embolic agent cast in the pedicles of intracranial major arterial feeders and capsule vessels. **l** The endovascular procedure achieved partial obliteration of the tumoral arterial feeders, leaving tenuous feeders from the anterior inferior cerebellar artery and occipital artery to be controlled microsurgically. **m**, **n**, **o** Follow-up magnetic resonance imaging at 1 month revealed gross-total resection of the tumor, resulting in postoperative subcutaneous effusion at the surgical site

A 73-year-old male presented with reduced flexibility in right limb movements for 3 months. The patient had an undergone an in the right hemisphere of the cerebellum 18 years prior and was pathologically diagnosed with hemangioblastoma. Physical examination revealed grade 4 muscle strength in the right limb, with positive Romberg’s test and alternating movement test results. MRI revealed a lobulated ring-shaped signal-occupying lesion approximately 51 mm × 52 mm × 51 mm in size in the right cerebellar hemisphere, with marked heterogeneous enhancement in contrast images and significant flow-void signs in T2-weighted images (Fig. 1af). Preoperative DSA showed hypervascular tumors fed by arterial pedicles, among which the majority were raised from the right posterior cerebellar artery, superior cerebellar artery, and right occipital artery, and the minority were raised from the anterioinferior cerebellar artery (Fig. 1g, h). A routine multidisciplinary preoperative discussion was held as a routine. Endovascular occlusion of arterial feeders is required to reduce blood loss during microsurgery, but tumor apoplexy edema is a concern. To meet the requirements of operative safety and efficiency, a one-stage hybrid operation, involving endovascular embolization to assist in occluding the pedicles of the major intracranial arterial feeders of the tumor, was performed, and the major feeder of the occipital artery was controlled via craniotomy. In such a case, care should be taken to avoid excessive immersion of the intratumoral vascular bed by embolic agents, which would make the tumor difficult to resect.

The patient underwent the endovascular procedure under general anesthesia in the supine position. A modified Seldinger puncture was performed on the right femoral artery, and a 6-F femoral sheath was inserted to establish access. A 6-F guiding catheter (ENVOY Guiding Catheter, Codman & Shurtleff, Inc., Miami Lake, FL, USA) was advanced under roadmap guidance and placed distal to the V1 segment of the right vertebral artery. Three-dimensional rotating angiography was performed to select the working position. A microguidewire (Synchro-10 0.010 inch × 200 cm, Stryker, Kalamazoo, MI, USA) and flow-directed microcatheter (Marathon, Covidien, Irvine, CA, USA) were advanced through the guiding catheter and superselected into the right posterioinferior cerebellar artery under the road map in the working position. The microcatheter was superselectively advanced to the distal end of the tumor-feeding artery under microroadmap guidance (Fig. 1i). An embolic agent (Onyx-18, Covidien, Irvine, CA, USA) was injected, and the casts were observed within the tumor capsule vessels and the main trunk of the feeding artery. Similar manipulations were performed on branches of the left superior cerebellar artery (Fig. 1j). In total, 2.0 ml of embolic agent was injected (Fig. 1k). Postembolization angiography via the guiding catheter revealed partial obliteration of the arterial feeders of the tumor, with the tenuous feeders from the anterior inferior cerebellar artery remaining (Fig. 1l). The occlusion rate of the target pedicle was 66.7%. The femoral sheath was preserved when intraoperative assistance was needed. The patient was subsequently placed in the left lateral decubitus position for microsurgery. The microsurgical resection was performed with a posterior midline approach. The tumor was found to have moderate hardness with relatively clear boundaries. The tumor was resected in pieces after easy separation of border capsular tissues. The microsurgery lasted for 7.25 h, with a bleeding volume of 500 ml. After tumor resection, the bone flap was removed to prevent unpredictable postoperative cerebral edema and hernia. The pathological diagnosis was hemangioblastoma (WHO I). The patient was discharged 10 days after the operation, with an unchanged mRS score of 2 points. Follow-up MRI at 1 month revealed complete resection of the tumor and postoperative subcutaneous effusion at the surgical site (Fig. 1 m-o).

## Discussion

The foundational principles of multimodality therapy for hypervascular CNS tumors were first articulated by Manelfe et al. in 1973 [11]. The use of preoperative embolization for tumor devascularization is widely accepted, with the aim of minimizing blood loss and reducing operating time, thereby increasing the safety and efficiency of the subsequent microsurgical resection procedure. However, despite widespread adoption of preoperative embolization in clinical practice, its definitive benefits remain debated. While some studies suggest it mitigates hemorrhagic risks and streamlines surgical workflows [12], others highlight inconsistent evidence regarding its effect on operative safety and complication rates [12, 13]. This uncertainty has fueled proposals for single-session combined endovascular and surgical interventions [14], aiming to capitalize on synergistic advantages while minimizing procedural delays. Against this backdrop, one-stage hybrid operations have emerged as a promising method, leveraging real-time integration of endovascular and microsurgical techniques in a single operative setting. Initial case series have demonstrated feasibility, though larger-scale validation is warranted to establish their role in standardizing care for hypervascular CNS tumors [9, 10]. In our study, patients who underwent the one-stage hybrid operation experienced a mean blood loss of 1,208 ± 1,193.6 mL (range: 150–4500 mL), which was relatively less than that of those who received multistage treatment and who had a mean blood loss of 1,651 ± 2,667.7 mL (range: 150–17000 mL). No embolization-related complications were observed in the one-stage hybrid operation group. Based on our experience, the endovascular intervention utilized in one-stage hybrid operations for treating hypervascular tumors of the CNS has distinct therapeutic objectives. This, in turn, leads to differences in role divisions, techniques, and materials compared with traditional staged treatment approaches.

Compared with multistage treatment, the cooperation mode of neurosurgical and endovascular procedures in one-stage hybrid operations reduces the burden of endovascular embolization. In traditional preoperative embolization, well-distributed embolic agents within the tumor vascular bed and capsular vessels could effectively devascularize the tumor and inhibit the recruitment of collateral arterial feeders, which could help decrease blood loss in subsequent microsurgical resection days later [15–18]. The well-distributed embolic agent obliterates the majority of arterial supplies and casts in the arterial branches of the tumor and normal vessels, which results in postembolization complications, such as cranial nerve palsy, intracranial hemorrhage, tumor ischemia, postembolization neurological syndromes, and subsequent hydrocephalus and hernia [19, 20]. However, the role of endovascular intervention has changed in one-stage hybrid operations, with this approach being used in various modes for cerebrovascular diseases. In the treatment of brain arteriovenous malformations, endovascular embolization has been used to control deep-site original arterial feeders to reduce intraoperative bleeding and eloquent cortical damage [21, 22]. The surgical treatment of hypervascular tumors shares a similar strategy with that for cerebrovascular diseases, in which endovascular embolizations act as intraluminal clips of accessible arterial pedicles. Achieving a high rate of devascularization is not the goal of endovascular embolization in one-stage hybrid operations. Easily accessible arterial pedicles are left for microsurgical/endoscopic control, as the cooperative mode of endovascular and microsurgical/endoscopic techniques in the treatment of brain arteriovenous malformation has been proposed. Although this treatment modality might reduce the effect of endovascular embolization in assisting with the devascularization of hypervascular tumors, it decreases the likelihood of damaging feeding artery branches and anastomotic branches, thereby lowering the incidence of surgical complications.

The change in the mode of action makes the requirements of the embolic agent different. In traditional preoperative embolization, the distribution and casting of embolic agents are the most important factors influencing the results of preoperative embolization. Therefore, liquid embolic agents with good dispersion properties, such as N-butyl cyanoacrylate, EVOH, and ethyl alcohol, have been developed [17, 23, 24]. Physicians are still making efforts to increase the dispersion of embolic agents, to downregulate their concentration, for example, ensuring that they are present in the vascular bed of the tumor as much as possible. Solid embolic agents, such as polyvinyl alcohol, microspheres, and GDCs, can only occlude the arterial pedicle of hypervascular tumors, which increases concerns about the recruitment of collateral arterial feeders during the interval between preoperative embolization and subsequent tumor resection [17, 25, 26]. Although novel solid embolic agents have been reported to achieve satisfactory embolization results [18], large-scale application is still a long way off. However, in one-stage hybrid operations, all the materials used to occlude the arterial pedicle could reduce the risk of injury to normal branch vessels and lower the incidence of ischemic complications. In addition, solid embolic agents enable the reuse of microcatheters, which is helpful in reducing the financial stress of patients. In this situation, temporary occlusion techniques, such as balloon catheters, could also be considered for controlling ongoing massive bleeding or preventing the occurrence of bleeding, as well as the introduction of new materials.

One-stage hybrid operations have driven advancements in treatment concepts and techniques for hypervascular central nervous system tumors. By relying on the new role and functions of interventional therapy within the one-stage hybrid surgery framework, as well as the synergistic interaction between interventional and surgical techniques, it is possible to effectively reduce surgical bleeding, protect neural functions, improve functional outcomes, and decrease surgical complications.

### Limitations of the study

The limitations of this study are as follows:

1. This study was retrospective in design and lacks a prospective experimental design, which results in certain limitations in the information collected.
2. The tumors in the cases studied were heterogeneous.

## Conclusion

One-stage hybrid operation is safe for the treatment of hypervascular CNS. A prospective study to evaluate its safety and efficacy compared with separate-stage treatment is needed.

## Data Availability

The datasets used and analyzed during the current study are available from the corresponding author upon reasonable request.

## Abbreviations

OR: Operating room
CNS: Central nervous system
CT: Computed tomography
MRI: Magnetic resonance imaging
EVOH: Ethylene-vinyl alcohol copolymer
GDC: Guglielmi detachable coil
DSA: Digital subtraction angiography
DNDs: Deterioration of neurological deficits
mRS: Modified Rankin Scale
